# Noise Minimization in Eukaryotic Gene Expression

**DOI:** 10.1371/journal.pbio.0020137

**Published:** 2004-04-27

**Authors:** Hunter B Fraser, Aaron E Hirsh, Guri Giaever, Jochen Kumm, Michael B Eisen

**Affiliations:** **1**Department of Molecular and Cell Biology, University of CaliforniaBerkeley, CaliforniaUnited States of America; **2**Department of Biological Sciences, Stanford UniversityStanford, CaliforniaUnited States of America; **3**Stanford Genome Technology CenterStanford, CaliforniaUnited States of America; **4**Genome Sciences Department, Genomics DivisionLawrence Berkeley National Laboratory, Berkeley, CaliforniaUnited States of America

## Abstract

All organisms have elaborate mechanisms to control rates of protein production. However, protein production is also subject to stochastic fluctuations, or “noise.” Several recent studies in Saccharomyces cerevisiae and Escherichia coli have investigated the relationship between transcription and translation rates and stochastic fluctuations in protein levels, or more generally, how such randomness is a function of intrinsic and extrinsic factors. However, the fundamental question of whether stochasticity in protein expression is generally biologically relevant has not been addressed, and it remains unknown whether random noise in the protein production rate of most genes significantly affects the fitness of any organism. We propose that organisms should be particularly sensitive to variation in the protein levels of two classes of genes: genes whose deletion is lethal to the organism and genes that encode subunits of multiprotein complexes. Using an experimentally verified model of stochastic gene expression in S. cerevisiae, we estimate the noise in protein production for nearly every yeast gene, and confirm our prediction that the production of essential and complex-forming proteins involves lower levels of noise than does the production of most other genes. Our results support the hypothesis that noise in gene expression is a biologically important variable, is generally detrimental to organismal fitness, and is subject to natural selection.

## Abstract 2

Analysis of gene expression data for nearly every gene in yeast provides evidence that random variation in the production rate of proteins could significantly affect the fitness of an organism

## Introduction

Stochasticity is a ubiquitous characteristic of life. Such apparent randomness, or “noise,” can be observed in a wide range of organisms, resulting in phenomena ranging from progressive loss of cell-cycle synchronization in an initially synchronized population of microbes to the pattern of hair coloration in female calico cats. An important source of stochasticity in biological systems is the random noise of transcription and translation, which can result in very different rates of synthesis of a specific protein in genetically identical cells in essentially identical environments (Elowitz et al. 2002; Ozbudak et al. 2002; Blake et al. 2003).

Understanding how stochasticity contributes to cellular phenotypes is important to developing a more complete picture of how cells work. Accordingly, noise in gene expression and other cellular processes has been a major focus of research for more than a decade. While several cases have been described where stochasticity is advantageous (e.g., phase variation in bacteria [Hallet 2001] and the lysis/lysogeny decision in phage lambda [Arkin et al. 1998]), it is expected that noise is not advantageous in most cellular processes, as precisely controlled levels of gene expression are presumably optimal (c.f. Barkai and Leibler 2000). However, whether noise in expression is of consequence to organismal fitness has not previously been investigated, despite the centrality of this question to our understanding of the role of noise in biological systems.

In this study, we investigate whether the differences in noise levels among genes are consistent with the hypothesis that noise in gene expression has been subject to natural selection to reduce its deleterious effects. We propose that random fluctuations in the expression levels of two groups of genes in yeast, essential genes and genes encoding protein complex subunits, should be particularly consequential for organismal fitness. If noise in gene expression is not an important factor to yeast—i.e., if the level of stochasticity experienced by yeast in gene expression is below that which would have negative consequences—then we would expect to see no difference in the randomness of expression in genes for which noisy expression is predicted to be relatively more or less deleterious. However, if stochasticity is an important variable on which natural selection has acted, we would expect to see the strongest signature of such selection in the expression of genes for which yeast are the most sensitive to randomness.

## Results

If deletion of a gene has only a small deleterious effect on the fitness of yeast, then random fluctuations in the amount of protein produced from that gene are likely to have a similarly small, or even smaller, impact. In contrast, the same fluctuations in the level of a protein essential for viability may have a profound effect on fitness; in the extreme, fluctuation to levels below that required for normal cellular function could compromise viability. Considering this predicted difference in the sensitivity of yeast to randomness in expression of essential versus relatively dispensable genes, we reasoned that if noise in gene expression is a biologically important variable, selection for reduction of stochasticity in expression levels would likely be stronger for essential genes than for nonessential ones.

A recent study linking noise in protein levels to transcription and translation rates in yeast (Blake et al. 2003) allows us to test this prediction. In the study, noise in the expression of a green fluorescent protein (GFP) reporter gene was measured by flow cytometry; stochasticity was measured as the amount of variation in GFP levels per cell in a population. Thus if all cells in a population had very similar levels of GFP, there was little noise in the production of the GFP. The effect of transcription and translation on noise levels was studied by independently varying these two parameters and measuring the resulting noise levels for a population of cells. This experimental approach, as well as a mathematical model of protein production (Blake et al. 2003), indicates that noise in protein production is maximized at intermediate levels of transcription (at approximately one-third of the maximal transcription rate of a gene, regardless of what that maximum is; see Materials and Methods), as well as at maximal levels of translation per mRNA molecule.

**Figure 1:**
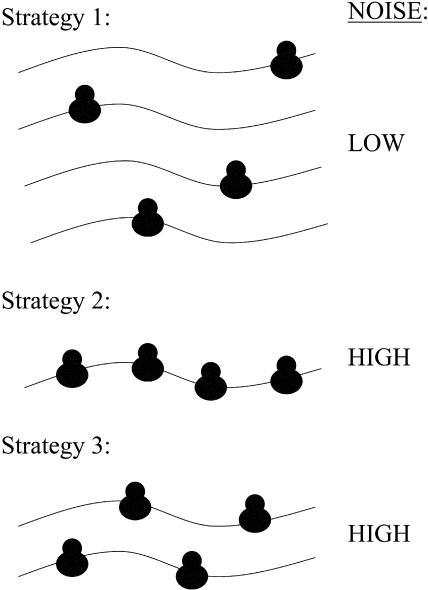
Strategies for Expression Three different strategies for achieving a given rate of protein production (four proteins will be produced in each case) and the amount of noise in expected to result from each strategy. Curved lines represent mRNA molecules, with ribosomes translating them; a larger number of mRNA molecules represents higher transcription, and a larger number of ribosomes per mRNA represents higher translation per mRNA.

To produce a given amount of any particular protein, yeast could adopt one of three qualitatively different strategies (Thattai and van Oudenaarden 2000) (Figure 1): (1) maximize transcription and minimize translation per mRNA, (2) maximize translation per mRNA and minimize transcription, or (3) employ intermediate levels of both transcription and translation per mRNA. Importantly, strategy 1 should result in less stochasticity than strategy 2 or 3. Strategy 2 is noisy due to the high translation, and strategy 3 is noisy due to both intermediate transcription and translation (the data currently available do not allow us to predict whether expression strategy 2 is more or less noisy than strategy 3). In contrast, noise is minimized at both transcription and translation steps for genes that exhibit strategy 1. Thus we predicted that if noise in protein production is an important factor in yeast, then genes that are essential for viability would be biased towards having high transcription rates and a low number of translations per mRNA.

To test this prediction, we estimated protein production rates (proteins/s; see Materials and Methods) for all yeast genes and asked whether essential genes tended to adopt strategy 1 more often than nonessential genes with similar protein production rates. It was critical to control for overall rates of protein production, as there is an overall correlation between a gene's dispensability (defined as the growth defect of a yeast strain missing that gene in rich glucose medium, i.e., an essential gene is indispensable) and its rate of protein production (Figure S1). This correlation between dispensability and the rate of protein synthesis may have nothing to do with stochasticity; most essential proteins may simply be needed in somewhat greater quantity than most nonessential proteins, so their genes must be more highly transcribed and/or translated. Since such a relationship could lead to an association between gene importance and the likelihood of adopting expression strategy 1, we employed two statistical methods to control for this possibility.

**Figure 2:**
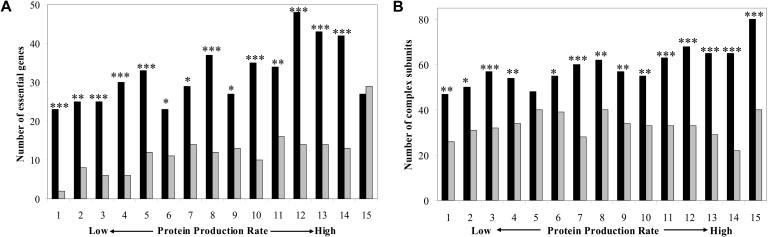
Essential Genes and Protein Complex Subunits Minimize Noise in Expression Binning analysis of (A) essential genes and (B) protein complex subunits. All genes for which transcription and translation rate data were available were separated into 15 bins by their protein production rate. Each bin was then separated into thirds by number of translations per mRNA. The two-thirds in each bin with the most extreme transcription and translation are shown: black bars are the number of each type of gene (essential or complex subunit) in the third of each bin with the lowest number of translations per mRNA and the highest transcription rate, and thus low noise; gray bars are the number of each type of gene in the third with the highest number of translations per mRNA and the lowest transcripton rate, and thus high noise. Bins are ordered by their rate of protein synthesis. The number of asterisks indicates the Fisher's exact test probability of observing the values for each bin under the null model of independence. *, *p* ≤ 0.02; **, *p* < 0.005; ***, *p* < 0.0005.

In the first of these two methods, we binned yeast genes by their protein production rate, so that all genes in each of 15 bins had approximately equal levels of protein production (see Table S1 for details). The genes in each bin could have achieved their similar protein production levels by any of the three strategies listed above; our prediction was that if noise in gene expression is relevant to yeast, then essential genes would be biased towards having the highest transcription and lowest translation per mRNA (strategy 1) in each bin. Indeed, this was confirmed by the data: when the genes within each bin were separated into thirds by their number of translations per mRNA, a larger number of essential genes were in the third with the lowest number of translations (low noise) than in the third with the highest number of translations (high noise) for all but one of 15 bins (Figure 2A). A Fisher's exact test (Sokal and Rohlf 1994) demonstrated that for all of the 14 bins with more essential genes in the low noise third than the high noise third, this difference was significant (*p* ≤ 0.02). Similar results were found when using different numbers of bins, when using halves or quartiles instead of thirds, or when separating bins by transcription rate instead of by number of translations per mRNA (data not shown). This result cannot be explained by the overall positive correlation between dispensability and rate of protein synthesis. (In the binning analysis, the third of each bin with the lowest translation rate had, on average, a slightly lower overall protein synthesis rate than the third with the highest translation rate [data not shown]; this bias is the opposite of what would be expected from the positive correlation between protein synthesis rate and fitness effect or protein complex membership, and thus it acts against our observed bias to make the results of this analysis conservative estimates of the true bias.)

Because binning genes still allows for a small amount of variability in protein production within each bin (see Table S1), we sought to control for protein production rate in another fashion as well. We employed partial correlation, a method that allows one to examine the relationship between two variables when other, possibly confounding, variables are statistically held constant (see Materials and Methods). The stochastic model of gene expression (Blake et al. 2003) led us to the prediction that, when protein production rate is controlled for, fitness effect (*f*, where *f* = 0 indicates no effect on growth when a gene is deleted, *f* = 1 indicates that a gene is essential, and 0 < *f* < 1 indicates a quantitative growth defect [Hirsh and Fraser 2001]) would correlate positively with transcription rate and negatively with translation rate per mRNA. Indeed, this is what we observed (*f* versus transcription [txn] rate | protein production rate, Spearman partial *r* = 0.282, *n* = 4,746, *p* = 10^−87^; *f* versus translations [tlns] per mRNA | protein production rate, Spearman partial *r* = −0.258, *n* = 4,746, *p* = 10^−75^). We also expected that the relationship between gene importance and implementation of the expression strategy that minimizes noise could additionally be seen by considering transcription rate and translation rate per mRNA together, as a ratio; a large ratio of transcription rate to translations per mRNA would indicate that transcripts are produced quickly but are translated slowly, corresponding to our expression strategy 1. Confirming this, the correlation between fitness effect and the ratio of transcription rate to translations per mRNA (controlling for protein production rate) is highly significant (*f* versus txn rate/tlns per mRNA | protein production rate, Spearman partial *r* = 0.275, *n* = 4,746, *p* = 10^−86^). Partial correlation analysis is thus in accordance with the trend illustrated in Figure 2A: essential genes preferentially use expression strategy 1, which minimizes stochasticity.

In addition to essential genes, genes whose protein products participate in stable protein complexes (“complex subunits”) would also be expected to exhibit sensitivity to randomness in expression: producing too little or too much of a single protein complex subunit can compromise the proper assembly of the entire complex and waste the energy invested in the production of the other complex subunits. In support of this, it has been found that both under- and overexpression of complex subunits is more likely to result in a reduced growth rate or inviability of yeast than is misexpression of other genes, and also that complex subunits tend to be more precisely coexpressed with other genes than noncomplex subunits (Papp et al. 2003). Using data from two high-throughput studies that identified proteins involved in stable complexes (Gavin et al. 2002; Ho et al. 2002), we assigned genes to two groups: those whose protein products were members of a stable complex found in either study and those whose protein products were not. (Since the protein complex data do not include all protein complexes, we expect that many protein complex subunits will not be classified as such in our list; this, as well as any false positives in the data, makes our results a conservative estimate the true strength of the effect.) We then performed the same binning analysis as described above, substituting our list of complex subunits for our list of essential genes. Again the prediction was confirmed: in all 15 bins, the third of the bin with the least translation per mRNA (and thus the lowest noise level) contained more complex subunits than the third with the most translation per mRNA (Figure 2B). The association between low translation per mRNA and protein complex membership was significant (Fisher's exact test, *p* ≤ 0.02) for all but one bin. As in Figure 2A, this result is robust with respect to the number of bins and the size of the divisions within bins (data not shown). Also as in Figure 2A, the bias is the opposite of that expected from the positive correlation between fitness effect and protein production rate; it is also the opposite of what would be the result of highly expressed genes being more likely to appear in the list of protein complex subunits than are poorly expressed genes. (It has been found that highly expressed genes are overrepresented in protein complex data [whether this is an experimental artifact or a true relationship is unclear; von Mering et al. 2002]; this would also act against our observed bias of complex subunits being overrepresented in the third with the lowest overall protein synthesis rate in each bin, thus making our results conservative.)

When we repeated the partial correlation analysis for complex subunits (genes were assigned a value of one if they were a complex subunit, zero if not), we found similar results. When total protein synthesis was controlled for with the partial correlation, complex subunits were more likely to have a high transcription rate (complex subunit versus txn rate | protein production rate, Spearman partial *r* = 0.203, *n* = 4,900, *p* = 10^−46^) and a low number of translations per mRNA (complex subunit versus tlns per mRNA | protein production rate, Spearman partial *r* = −0.200, *n* = 4,900, *p* = 10^−46^). Using the ratio of transcription rate to translations per mRNA also yielded similar results (complex subunit versus txn rate/tlns per mRNA | protein production rate, Spearman partial *r* = 0.220, *n* = 4,900, *p* = 10^−56^). Thus, partial correlations confirm the finding illustrated in Figure 2B.

Since proteins that participate in many protein–protein interactions are more likely to be essential (Jeong et al. 2001; Fraser et al. 2002), it was not immediately clear whether protein fitness effect and membership in a multiprotein complex are independently associated with the expression strategy that minimizes stochastic fluctuations. To address this question, we calculated the partial correlation between fitness effect and the ratio of transcription rate to translations per mRNA, while controlling for both protein production rate and protein complex membership. Likewise, we calculated the correlation between protein complex membership and the ratio of transcription rate to translation rate per mRNA while controlling for both protein production rate and fitness effect. The two partial correlations were both quite significant (*f* versus txn rate/tlns per mRNA | protein production rate, complex membership: Spearman partial *r* = 0.227, *n* = 4,746, *p* = 10^−57^; complex membership versus txn rate/tlns per mRNA | protein production rate, *f:* Spearman partial *r* = 0.147, *n* = 4,746, *p* = 10^−24^), suggesting that fitness effect and protein complex membership are independently associated with the expression strategy that minimizes stochastic fluctuation. (However, the relative strengths of the partial correlations cannot be interpreted as their true relative contributions because of the differing quality of fitness effect and protein complex membership data.) Repeating the partial correlations above with either transcription rate or translations per mRNA in place of their ratio gave significant partial correlations with both fitness effect and protein complex membership as well (data not shown).

The hypothesis that genes of large fitness effect are under stronger selection to reduce stochastic fluctuation in expression provides an explanation for a previously observed, but as yet unexplained, correlate of protein evolutionary rate. Pal et al. (2001) noted a weak but significant negative correlation (*r* = −0.11, *p* = 10^−9^) between an mRNA's rate of decay and the evolutionary rate of the protein it encodes. This correlation was surprising, as it is precisely the opposite of what one would expect if the relationship between the rates of mRNA decay and protein evolution were mediated by the level of expression: slow decay would result in increased expression, which is known to be associated with slow evolution (Pal et al. 2001). Thus, we would expect a positive correlation between rates of mRNA decay and protein evolution, not the negative one that is observed. However, under the present hypothesis that relatively important genes are under stronger selection to reduce noise, the relationship between mRNA decay and protein evolutionary rate is interpretable. Both genes of large fitness effect and genes that encode protein complex subunits are known to evolve slowly (Hirsh and Fraser 2001; Fraser et al. 2002; Jordan et al. 2002). (While the reason why genes of large fitness effect evolve slowly has been debated [Hirsh and Fraser 2003; Pal et al. 2003], the presence of the correlation has not been disputed, and it can be seen to be much stronger than previously reported when using more accurate fitness effect and evolutionary rate data [data not shown]). Here we have shown that they are also associated with a strategy of expression that maximizes the rate of transcription and minimizes the number of translations per mRNA. Given a desired rate of protein production, one way to maximize transcription rate while minimizing the number of translations per mRNA is to maximize the mRNA decay rate. Thus, we would expect rapid mRNA decay among essential genes and protein complex subunits, both of which evolve slowly, yielding the observed negative correlation between the rates of mRNA decay and protein evolution. In support of this prediction, both essential genes and protein complex subunits have substantially shorter mRNA half-lives than the rest of the genome (e.g., mRNA half-lives of nonessential genes are 32% longer than those of essential genes, and the bias remains when controlling for protein production rate; *p* i= 10^−36^ by the Wilcoxon test [Sokal and Rohlf 1994]).

## Discussion

We found that noise in protein production is minimized in genes for which it is likely to be most harmful, specifically essential genes and genes encoding protein complex subunits. This finding supports the hypothesis that noise in gene expression is generally deleterious to yeast.

Yeast appear to control the noise in their gene expression at both transcriptional and translational levels preferentially for some genes; however, this noise minimization is not without a cost, as the high transcription and high mRNA decay rates that are needed to minimize noise are energetically expensive and are thus expected to be advantageous only when the benefit of reducing noise in a particular gene's expression outweighs this cost (Thattai and van Oudenaarden 2000). Protein degradation rates may also play a role in controlling noise, but this cannot be tested until genome-wide protein degradation rates have been measured.

As is the case with many genome-wide studies, it is possible that a hidden variable could bias our results. For example, it is possible that essential genes and genes encoding protein complex subunits tend to have high transcription and low translation for reasons unrelated to noise minimization. However, until such a reason is identified, the most parsimonious interpretation of our results is that yeast adaptively minimize noise in the expression of certain genes.

As genome-wide transcription and translation rate data become available for other organisms, it will be interesting to see if the apparent tendency to minimize noise in the expression of important genes extends to organisms other than yeast. Considering that several anecdotal examples of indispensable genes with unusually low translation rates, and thus low noise in expression, have already been noted in Escherichia coli (Ozbudak et al. 2002), this could well be the case.

## Materials and Methods

### 

#### Functional genomic data sources

Transcription rates were calculated from mRNA abundances and decay rates in log-phase yeast growing in rich glucose medium (Wang et al. 2002) according to the steady-state equation *R* = −ln(0.5) * *A*/*T*, where *R* is transcription rate, *A* is mRNA abundance, and *T* is mRNA half-life. Translation rates per mRNA in rich glucose medium were calculated from ribosome occupancy data by Arava et al. (2003); specifically, ribosome density per mRNA present in the polysome fraction was multiplied by the fraction of each mRNA that was found in the polysome fraction to estimate the average ribosome density for all copies of each mRNA in a cell. This density is equivalent to a relative translation rate, assuming that the speed at which ribosomes produce proteins is constant over different mRNAs. An estimate of the actual translation rate was found by multiplying the relative translation rates by a constant: the speed of translation, which is approximately ten amino acids/s (Arava et al. 2003). Protein production rate (proteins/s) was then calculated by multiplying translation rate per mRNA with mRNA abundance. Note that the protein production rate can also be represented as the product of transcription rate and number of translations per mRNA. It is this latter variable that was used to separate each bin into thirds in Figure 2, since it is thought to be more directly relevant to noise in protein production than related quantities such as translation rate per mRNA (Berg 1978); the variable was calculated by dividing protein production rate by transcription rate for each gene. However, bins could also be separated into thirds by transcription rate, transcript abundance, or translation rate per mRNA, all yielding similar results (data not shown).

Fitness effect ranks were calculated from 12 replicate growth experiments for all viable homozygous yeast deletion strains in rich glucose medium; growth experiments were conducted using the method described in Giaever et al. (2004). The logarithms of deletion strain tag fluorescence intensities on high-density oligonucelotide microarrays for each growth time course were fitted to a linear model that accounted for time-course-specific effects and variable initial strain concentrations. The slope of the linear regression was then used as the relative growth rate for each strain.

#### Estimates of percent induction levels

Blake et al. (2003) showed for two different promoters in yeast, as well as in their mathematical model, that noise due to transcription peaked at approximately one-third of maximal transcriptional induction. Importantly, one of their promoters (P_ADH1*_) was 10-fold weaker than the other two at full induction, but all three showed very similar relationships between noise strength and percent transcriptional induction. Since we do not have genome-wide data for the percent induction for genes in rich glucose medium (or any other environment), in our analysis we make the assumption that the promoters of more highly transcribed genes tend to be at higher percent induction levels. While this certainly does not hold for all genes, we believe that it is a reasonable approximation for most genes.

#### Partial correlations

Partial correlations were calculated as described by Sokal and Rohlf (1994). Briefly, let *r_XY_* be the correlation coefficient between variables *X* and *Y.* To control for a third variable *Z,*



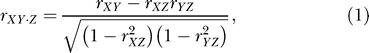



To assess the significance of the partial correlation, it is transformed to be distributed according to a Student's *t* distribution, by the equation



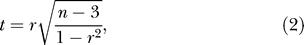



The two-sided *p*-value can then be calculated according to where the *t*-value falls with respect to its expected distribution.

## Supporting Information

Figure S1 The Relationship between Fitness Effect and Protein Production Rate Fitness effect ranks are shown on the y-axis (the large number of points at 519.5 are the essential genes, with fitness effect = 1). Protein production rate (proteins/s) is shown on the x-axis. The Spearman rank correlation coefficient is r = –0.202 (p = 10^–49^).
(316 KB PPT).

Table S1 Details of the Protein Production Rates (Protein/s) within Each Bin from Figure 2 (37 KB DOC).

## Abbreviations

GFP: green fluorescent protein
tln: translation
txn: transcription
