# Supplementary figures and images for: Noise Minimization in Eukaryotic Gene Expression
Source: PLoS Biol. 2004 Apr 27;2(6):e137. doi: 10.1371/journal.pbio.0020137 (PMC400249; doi:10.1371/journal.pbio.0020137)

## Slide 1
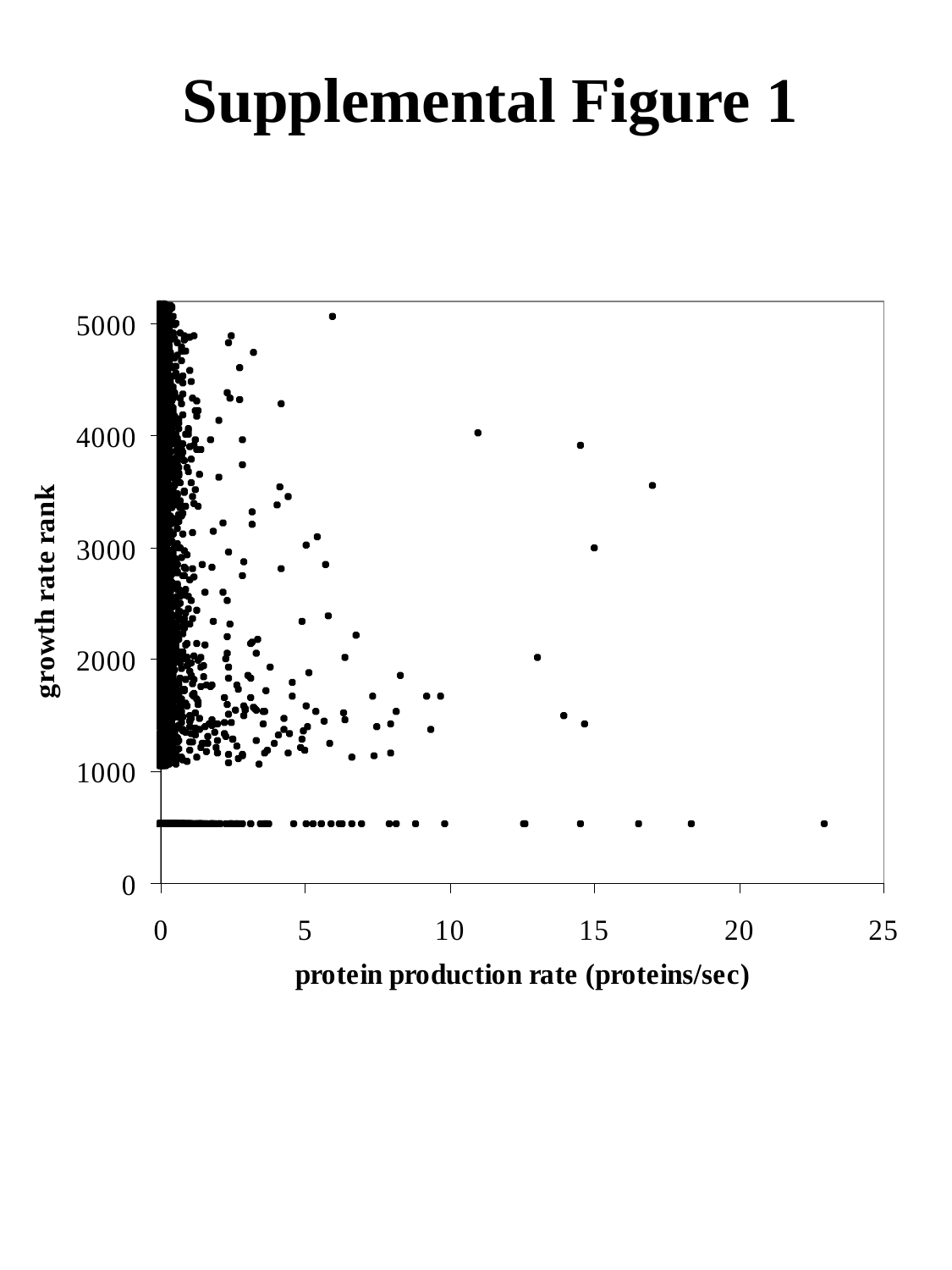

Supplemental Figure 1

Supplement: Figure S1 — Fitness effect ranks are shown on the y-axis (the large number of points at 519.5 are the essential genes, with fitness effect = 1). Protein production rate (proteins/s) is shown on the x-axis. The Spearman rank correlation coefficient is r = –0.202 (p = 10–49). (316 KB PPT). [file pbio.0020137.sg001.ppt]
