# Supplementary material for: Noise Minimization in Eukaryotic Gene Expression
Source: PLoS Biol. 2004 Apr 27;2(6):e137. doi: 10.1371/journal.pbio.0020137 (PMC400249; doi:10.1371/journal.pbio.0020137)
Supplement: Table S1 — (37 KB DOC). [file pbio.0020137.st001.doc]

**Table S1.** Details of the Protein Production Rates (Proteins/S) within Each Bin from Figure 2

| Bin | Minimum | Maximum | Mean | Standard Deviation |
| --- | --- | --- | --- | --- |
| 1 | 0 | 0.0157 | 0.0103 | 0.0037 |
| 2 | 0.0157 | 0.0237 | 0.0198 | 0.0023 |
| 3 | 0.0238 | 0.0329 | 0.0286 | 0.0026 |
| 4 | 0.033 | 0.0452 | 0.0387 | 0.0035 |
| 5 | 0.0453 | 0.0611 | 0.0529 | 0.0048 |
| 6 | 0.0612 | 0.0784 | 0.0692 | 0.005 |
| 7 | 0.0784 | 0.1006 | 0.0895 | 0.0063 |
| 8 | 0.1008 | 0.1277 | 0.114 | 0.0082 |
| 9 | 0.1279 | 0.1602 | 0.1439 | 0.0089 |
| 10 | 0.1604 | 0.2014 | 0.1791 | 0.0116 |
| 11 | 0.2014 | 0.2602 | 0.2289 | 0.0164 |
| 12 | 0.2602 | 0.3419 | 0.301 | 0.0237 |
| 13 | 0.3422 | 0.4877 | 0.4019 | 0.0409 |
| 14 | 0.4882 | 0.8848 | 0.6458 | 0.109 |
| 15 | 0.8855 | 13.0892 | 2.7383 | 2.2497 |

Listed are the minimum, maximum, mean, and standard deviation of protein production rates within each of the 15 bins.
